# Reprogramming progeria fibroblasts re‐establishes a normal epigenetic landscape

**DOI:** 10.1111/acel.12621

**Published:** 2017-06-08

**Authors:** Zhaoyi Chen, Wing Y. Chang, Alton Etheridge, Hilmar Strickfaden, Zhigang Jin, Gareth Palidwor, Ji‐Hoon Cho, Kai Wang, Sarah Y. Kwon, Carole Doré, Angela Raymond, Akitsu Hotta, James Ellis, Rita A. Kandel, F. Jeffrey Dilworth, Theodore J. Perkins, Michael J. Hendzel, David J. Galas, William L. Stanford

**Affiliations:** ^1^ The Sprott Centre for Stem Cell Research Regenerative Medicine Program Ottawa Hospital Research Institute Ottawa Ontario Canada K1H 8L6; ^2^ Department of Cellular and Molecular Medicine University of Ottawa Ottawa Ontario Canada; ^3^ Pacific Northwest Diabetes Research Institute 720 Broadway Seattle WA 98103 USA; ^4^ Cross Cancer Institute and the Department of Experimental Oncology Faculty of Medicine and Dentistry University of Alberta Edmonton Alberta Canada T6G 1Z2; ^5^ Ottawa Bioinformatics Core Facility The Sprott Centre for Stem Cell Research Ottawa Hospital Research Institute Ottawa Ontario Canada K1H 8L6; ^6^ Department of Chemical Engineering University of Toronto Toronto Ontario Canada; ^7^ Center for iPS Cell Research and Application (CiRA) Kyoto University Kyoto Japan; ^8^ Program in Developmental and Stem Cell Biology The Hospital for Sick Children Toronto Ontario Canada; ^9^ Department of Molecular Genetics University of Toronto Toronto Ontario Canada; ^10^ Pathology and Experimental Medicine Mount Sinai Hospital Toronto Ontario Canada; ^11^ Department of Biochemistry, Microbiology and Immunology University of Ottawa Ottawa Ontario Canada; ^12^ Ottawa Institute of Systems Biology Ottawa Ontario Canada

**Keywords:** aging, epigenetics, induced pluripotent stem cells, Lamin A, lamina, progeria, reprogramming

## Abstract

Ideally, disease modeling using patient‐derived induced pluripotent stem cells (iPSCs) enables analysis of disease initiation and progression. This requires any pathological features of the patient cells used for reprogramming to be eliminated during iPSC generation. Hutchinson–Gilford progeria syndrome (HGPS) is a segmental premature aging disorder caused by the accumulation of the truncated form of Lamin A known as Progerin within the nuclear lamina. Cellular hallmarks of HGPS include nuclear blebbing, loss of peripheral heterochromatin, defective epigenetic inheritance, altered gene expression, and senescence. To model HGPS using iPSCs, detailed genome‐wide and structural analysis of the epigenetic landscape is required to assess the initiation and progression of the disease. We generated a library of iPSC lines from fibroblasts of patients with HGPS and controls, including one family trio. HGPS patient‐derived iPSCs are nearly indistinguishable from controls in terms of pluripotency, nuclear membrane integrity, as well as transcriptional and epigenetic profiles, and can differentiate into affected cell lineages recapitulating disease progression, despite the nuclear aberrations, altered gene expression, and epigenetic landscape inherent to the donor fibroblasts. These analyses demonstrate the power of iPSC reprogramming to reset the epigenetic landscape to a revitalized pluripotent state in the face of widespread epigenetic defects, validating their use to model the initiation and progression of disease in affected cell lineages.

## Introduction

Hutchinson–Gilford progeria syndrome (HGPS) is a rare and debilitating disease that affects one in four million live births (Merideth *et al*., 2008). This segmental aging syndrome is usually diagnosed within the first 2 years of life, and is characterized by a rapid progression of aging‐related tissue pathologies, including osteoporosis, scleroderma, lipodystrophy, alopecia, hearing loss, and arteriosclerosis (Capell & Collins, 2006; Merideth *et al*., 2008). Hutchinson–Gilford progeria syndrome children begin to exhibit widespread atherosclerosis by 5 years of age, which progresses rapidly and usually results in death by myocardial infarction or stroke in the teenage years (Merideth *et al*., 2008). The pathology of atherosclerotic plaques in progeria patients shows they are similar to those of aged individuals (Olive *et al*., 2010). Mutations within the *Lamin A/C* gene are the primary cause of HGPS (De Sandre‐Giovannoli *et al*., 2003; Eriksson *et al*., 2003; Csoka *et al*., 2004). The most common HGPS mutation is a C to T point mutation in exon 11 at a cryptic splice site in Lamin A (G608G), leading to the activation of this splice site to generate an alternatively spliced Lamin A isoform encoding an internal 50‐amino acid deletion (McClintock *et al*., 2007). The shorter Lamin A protein fails to mature correctly, resulting in a farnesylated product termed Progerin. Accumulation of Progerin within the nuclear lamina is also detected in the normative aging population, demonstrating that the cryptic splice site can also be utilized in the general population (McClintock *et al*., 2007). Thus, it has been hypothesized that Progerin accumulation contributes to the pathology of aging, leading to the use of the HGPS as a general model for aging.

Hutchinson–Gilford progeria syndrome is classified as a laminopathy, a heterogeneous group of diseases originating from structural defects in the nuclear lamina (Camozzi *et al*., 2014). The nuclear lamina is a protein‐based scaffold underlying the inner nuclear membrane, primarily composed of any of the four type V intermediate filament lamin proteins. In addition to providing structural and mechanical support for the nucleus, the lamins regulate a host of biological functions including cell signaling, transcriptional regulation, replication, and chromatin organization (Kennedy *et al*., 2000; Shumaker *et al*., 2006). At the molecular level, HGPS patient fibroblasts demonstrate altered nuclear architecture, activation of the DNA damage response (DDR), loss of epigenetic inheritance marked by loss of peripheral heterochromatin, altered gene expression and premature senescence (Eriksson *et al*., 2003; Goldman *et al*., 2004; Liu *et al*., 2006; Scaffidi & Misteli, 2006).

Much of our understanding of the molecular and cellular features of HGPS has come from the analyses of patient dermal fibroblasts or overexpression of Progerin in genotypically normal human cells (Eriksson *et al*., 2003; Goldman *et al*., 2004; Scaffidi & Misteli, 2006; Shumaker *et al*., 2006). However, the cellular function and epigenetic landscape of fibroblasts are starkly different from the vascular cells that become atherosclerotic and are responsible for the fatal cardiovascular and cerebrovascular disease or the chondrocytes that deteriorate and lead to osteoarthritis in patients with HGPS. Furthermore, patient samples within various biobanks have limited remaining replicative lifespan and, as a population, already demonstrate molecular phenotypes of HGPS, thereby limiting the ability to dissect the molecular initiation and progression of the disease. While overexpression of Progerin can be used to model the effects of Progerin in a tissue‐specific manner and monitor disease initiation and progression, artificially altering the stoichiometry of Progerin and Lamin A has its own limitations. In contrast, patient‐derived induced pluripotent stem cells (iPSCs) (Takahashi *et al*., 2007) offer an experimental approach to dissect the molecular mechanisms associated with Progeria in a tissue‐specific manner. Furthermore, the majority of Progeria studies using patient dermal fibroblasts were performed based on the assumption that Progerin accumulation directly leads to DNA damage and loss of heterochromatin, but this has not been directly tested. Providing that reprogramming represses Progerin expression and reverts HGPS cells to a pluripotent state with a rescued normal epigenetic landscape, HGPS iPSCs have the capacity to allow longitudinal analysis of the progression of HGPS from the initiation of Progerin expression to the onset of downstream nuclear defects. Here, we report a resource of iPSC lines generated from three patients with HGPS and three nonaffected individuals, including a trio of both genotypically normal parents and the affected HGPS child. Whole‐genome transcriptional and epigenetic profiling as well as electron microscopy verified that the library of iPSC lines are highly similar to normal embryonic stem cells (ESCs), demonstrating that epigenetic reprogramming can successfully generate normal iPSCs from epigenetically compromised dermal fibroblasts.

## Results

### HGPS fibroblasts used for reprogramming exhibit nuclear abnormalities and premature senescence in culture

**Figure 1:**
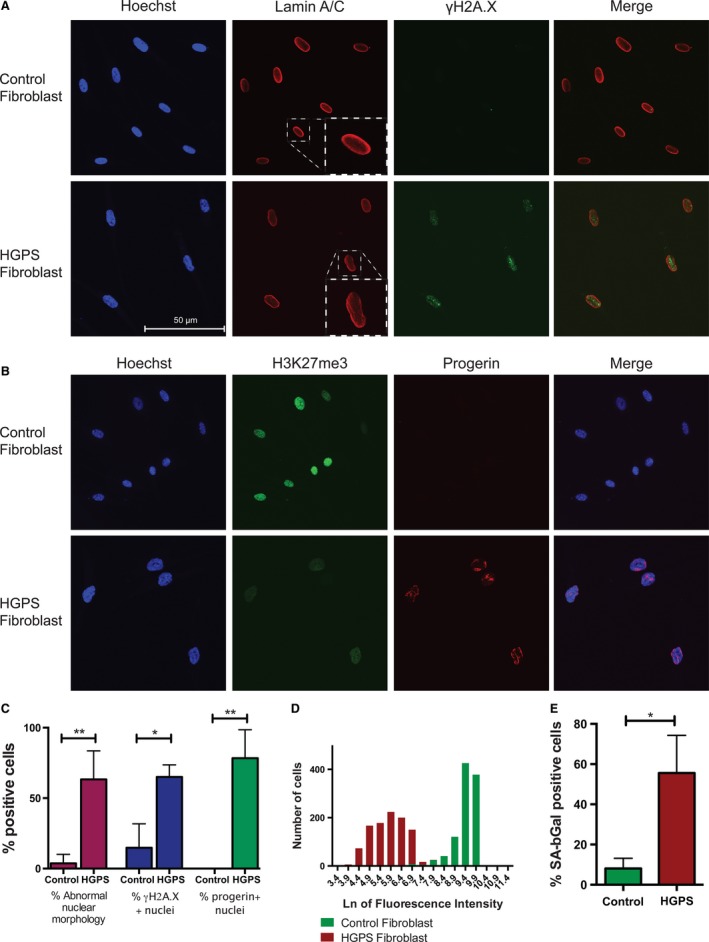
Hutchinson–Gilford progeria syndrome (HGPS) fibroblasts exhibit numerous nuclear defects and undergo premature senescence. (A) Immunofluorescence analysis of Lamin A/C reveals abnormalities in nuclear morphology in HGPS fibroblasts. HGPS fibroblasts also have increased γH2A.X signal compared to control fibroblasts. Nuclei are shown in blue. (B) Immunofluorescence analysis demonstrates reduced H3K27me3 marks and expression of Progerin in HGPS fibroblast nuclei. (C) Quantification of fibroblasts demonstrating increased abnormal nuclear morphology, accumulation of DNA damage, and expression of Progerin in HGPS fibroblasts compared to control fibroblasts. Results were assessed by immunofluorescence microscopy and ImageJ software. (D) High‐content imaging and fluorescence quantification on a natural log scale showing a loss of H3K27me3 in HGPS fibroblast nuclei compared to control fibroblasts. (E) An increased number of HGPS fibroblasts are positive for senescence‐associated β‐galactosidase activity compared to control fibroblasts, as assessed by flow cytometry. *indicates *P* value < 0.05 and ** indicates *P* value < 0.01 measured with Student's *t*‐test.

Along with accumulation of Progerin, HGPS fibroblasts have been reported to display several cellular defects including dysmorphic nuclei (Eriksson *et al*., 2003; Goldman *et al*., 2004) and increased DNA damage (Liu *et al*., 2006; Scaffidi & Misteli, 2006). Altered expression of various nuclear proteins and chromatin marks such as reduced heterochromatin‐specific histone 3 lysine 27 trimethylation (H3K27me3) (Goldman *et al*., 2004; Shumaker *et al*., 2006) has also been reported in HGPS fibroblasts. To assess the disease state in our patient samples used for reprogramming and the capacity of reprogramming to re‐establish a normal pluripotent epigenetic landscape in iPSCs, we first focused on characterizing these hallmark defects in our HGPS patient fibroblasts. All analyses were completed on three HGPS patient fibroblasts with the predominant G608G *LMNA* mutation (HGADFN167, HGADFN003, AG01972) and compared with fibroblast cultures from three unaffected individuals (HGFDN168, HGMDFN090, BJ) (Table 1). Importantly, the fibroblasts reprogrammed and characterized included a familial trio of two unaffected parents (HGFDN168, HGMDFN090) and one affected progeny HGADFN167. This trio provides a unique opportunity to directly compare iPSCs from related individuals. To characterize nuclear defects in the patient fibroblasts, we performed immunofluorescence staining for Lamin A and objectively quantified nuclear shape using an ImageJ analysis application. Significantly more HGPS fibroblasts displayed nuclei with irregular morphology, compared to normal fibroblasts (63% vs. 11%, respectively) (Fig. 1A,C). Additionally, significantly more HGPS fibroblasts stained positive for γH2A.X, a marker of the DDR (Fig. 1A,C). Both nuclear defects and increased activation of the DDR suggest these HGPS patient fibroblasts at the stage of reprogramming are phenotypically similar to other reported HGPS fibroblast lines (Eriksson *et al*., 2003; Liu *et al*., 2006).

**Table 1:** Induced pluripotent stem cells derived from three patients with Hutchinson–Gilford progeria syndrome (HGPS) and three unaffected controls. Two clones were used from each individual. Patient HGFDFN168 and HGMDFN090 are the unaffected parents of HGPS patient HGADFN167

Patient	Cell line	iPS clones
HGPS	HGADFN167	1J, 1Q
	HGADFN003	1B, 1C
	AG01972	1B, 1D
Control	HGMDFN090	1B, 1C
	HGFDFN168	1D2, 1P
	BJ	1C, 1D

In the HGPS fibroblasts used for reprogramming, 78% of cells expressed detectable levels of nuclear Progerin, in contrast to our normal fibroblasts, which contain no detectible Progerin‐positive cells (Fig. 1B,C). Previous analyses have demonstrated that Progerin accumulation is associated with alterations in repressive histone marks, including H3K27me3 (Shumaker *et al*., 2006). High‐content imaging showed a significant decrease in H3K27me3 in HGPS fibroblast nuclei compared to normal fibroblasts (Fig. 1B,D). These data indicate a loss of facultative heterochromatin and abnormal epigenetic landscape in the HGPS fibroblast cultures. Reprogramming requires active cell proliferation; however, HGPS fibroblasts are known to express senescence‐associated β‐galactosidase (Goldman *et al*., 2004). Using flow cytometry, we observed more than 50% of HGPS fibroblasts expressing senescence‐activated β‐galactosidase compared to < 10% in control fibroblasts (Fig. 1E). Together, these data demonstrate that the HGPS patient fibroblasts we used for reprogramming are similar to other HGPS fibroblast lines, displaying nuclear defects, chromatin abnormalities, and premature senescence.

### Reprogramming HGPS and unaffected fibroblasts to iPSCs

**Figure 2:**
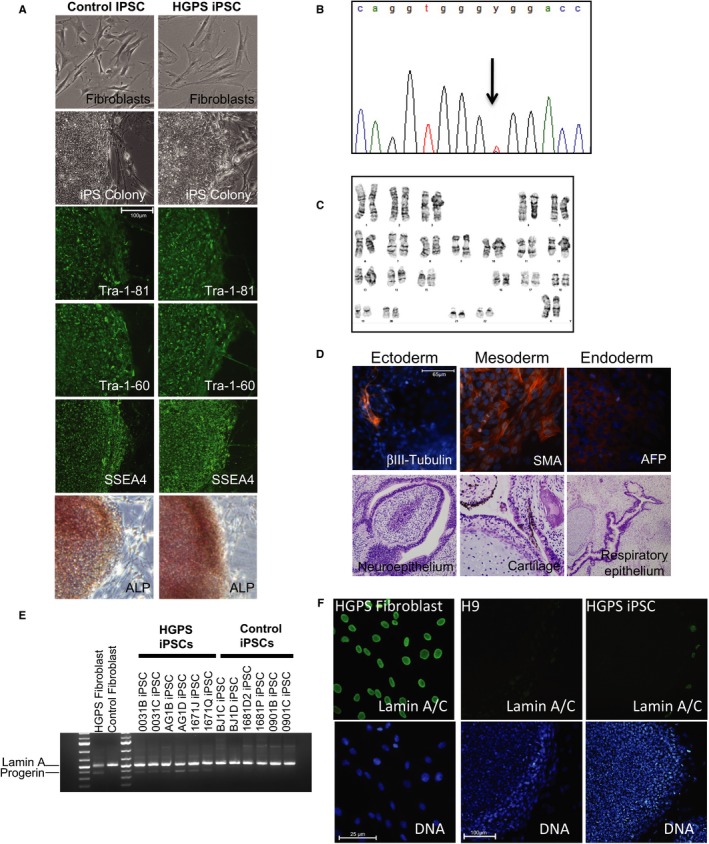
Induced pluripotent stem cells (iPSCs) derived from patients with HGPS and control individuals’ fibroblasts are pluripotent. (A) iPSC colonies demonstrating normal pluripotent stem cell colony morphology were derived from both HGPS and unaffected control fibroblasts following retroviral reprogramming and expressed markers of pluripotency, including TRA‐1‐81, TRA‐1‐60, SSEA4, and alkaline phosphatase (ALP). Expression levels of pluripotency markers were similar in HGPS and unaffected controls. (B) All HGPS patients carry the G608G mutation in Lamin A/C demonstrated by sequencing fibroblast and iPSC clones. Arrow indicates mutated base. (C) Karyotyping of both control and HGPS iPSCs reveals normal karyotype with no gross chromosomal abnormalities following reprogramming. (D) Top row, HGPS iPSCs differentiated *in vitro* generated cells from all three germ layers, exemplified by βIII‐tubulin (ectoderm), smooth muscle actin (SMA, mesoderm), and alpha‐fetoprotein (AFP, endoderm) expression. Bottom row, *in vivo* differentiation by teratoma formation confirms that HGPS iPSCs can differentiate into tissues from all three germ layers. Representative H&E‐stained micrographs are shown. (E) The mRNA transcripts of Lamin A and its truncated form (Progerin) are expressed in HGPS fibroblasts. In HGPS iPSCs, both mRNA transcripts are expressed, with Progerin being expressed at low levels. Progerin transcripts are not detected in normal fibroblasts and their derived iPSC clones. (F) Lamin A is expressed in HGPS fibroblasts but is downregulated in iPSC colonies following reprogramming, with expression being observed only in differentiated cells on the periphery of the colonies, comparable to control human embryonic stem cells (H9).

Based on the characterization of pathological hallmarks described above, HGPS fibroblast cultures present the full set of molecular phenotypes of Progeria and are therefore not suitable to perform longitudinal analyses to observe the onset of pathology, which is required to determine the direct cause of DNA damage or loss of epigenetic inheritance in Progeria cells. To generate a research tool that could be used to examine HGPS progression from the initiation stages of the disease, we reprogrammed fibroblasts from patients and unaffected controls into iPSCs. From the HGPS fibroblasts, we derived six iPSC clones, with two clones from each patient (Table 1). In addition, we generated six control iPSC clones derived from three normal fibroblast lines (Table 1). We observed no differences in reprogramming efficiency, generating similar numbers of iPSC colonies regardless of the genotype of the fibroblasts or the severity of the nuclear defects or other HGPS phenotypes. Both normal and HGPS iPSCs were morphologically indistinguishable from human ESCs (hESCs). All clones expressed pluripotency markers Tra‐1‐60, Tra‐1‐81, SSEA‐4, and alkaline phosphatase at levels comparable to hESCs (Figs 2A and S1B,C). We also confirmed the G608G mutation within our HGPS iPSC lines using Sanger sequencing, verifying the expected C to T mutation in all HGPS clones (Fig. 2B), indicating that we had derived pluripotent cells from affected patient cells. All normal and HGPS‐derived iPSC clones also displayed normal karyotypes (Fig. 2C). Each of these clones was assessed for pluripotency by *in vitro* and *in vivo* differentiation assays. Differentiation *in vitro* through embryoid body (EB) formation generated cells representative of each of the three germ layers, exemplified by the expression of markers of ectoderm (βIII‐tubulin), mesoderm [smooth muscle actin (SMA)], and endoderm (α‐fetoprotein, AFP). Additionally, all iPSC clones formed teratomas *in vivo*, comprised of tissues representative of all three germ layers, including neuroepithelium, cartilage, and respiratory epithelium (Figs 2D and S1D). Together, these *in vitro* and *in vivo* differentiation data demonstrate that each iPSC clone derived from normal and HGPS patients are pluripotent, enabling them to be differentiated into relevant cell types for modeling HGPS.

### Lamin A is downregulated following reprogramming

Previous reports have established that Lamin A protein is not expressed in undifferentiated pluripotent stem cells and that the *LMNA* transcript is downregulated during reprogramming (Rober *et al*., 1989; Constantinescu *et al*., 2006). We sought to confirm this by examining the expression of Lamin A in the HGPS iPSC clones at both mRNA and protein levels. As the G608G mutation activates a cryptic splice site and the aberrant removal of 150 nucleotides in exon 11 (De Sandre‐Giovannoli *et al*., 2003; Eriksson *et al*., 2003), we used primers that detect both the full‐length and the truncated mRNA produced from the *LMNA* gene. This allows detection of both the *LMNA* and *Progerin* transcript. RT–PCR analyses using these primers confirm both *LMNA* and *Progerin* transcripts are expressed in HGPS patient fibroblasts, while only *LMNA* transcripts are expressed in normal fibroblasts. After reprogramming, HGPS iPSCs expressed both *LMNA* and *Progerin* transcripts at low levels, while only *LMNA* transcript was detected in normal iPSCs (Fig. 2E). At the protein level, while Lamin A is expressed in fibroblasts, only differentiated cells at the edge of the iPSC colony had detectable Lamin A expression, which was not observed in the highly compact, undifferentiated region of the colonies (Figs 2F and S1B). In contrast, no Progerin protein was detectable in iPSCs under pluripotent culture conditions (Fig. 7E–F). This demonstrates that *LMNA* expression in patient and normal iPSCs was downregulated during reprogramming and Progerin expression is extinguished in undifferentiated HGPS iPSC clones.

### A normal nuclear morphology is restored following reprogramming

**Figure 3:**
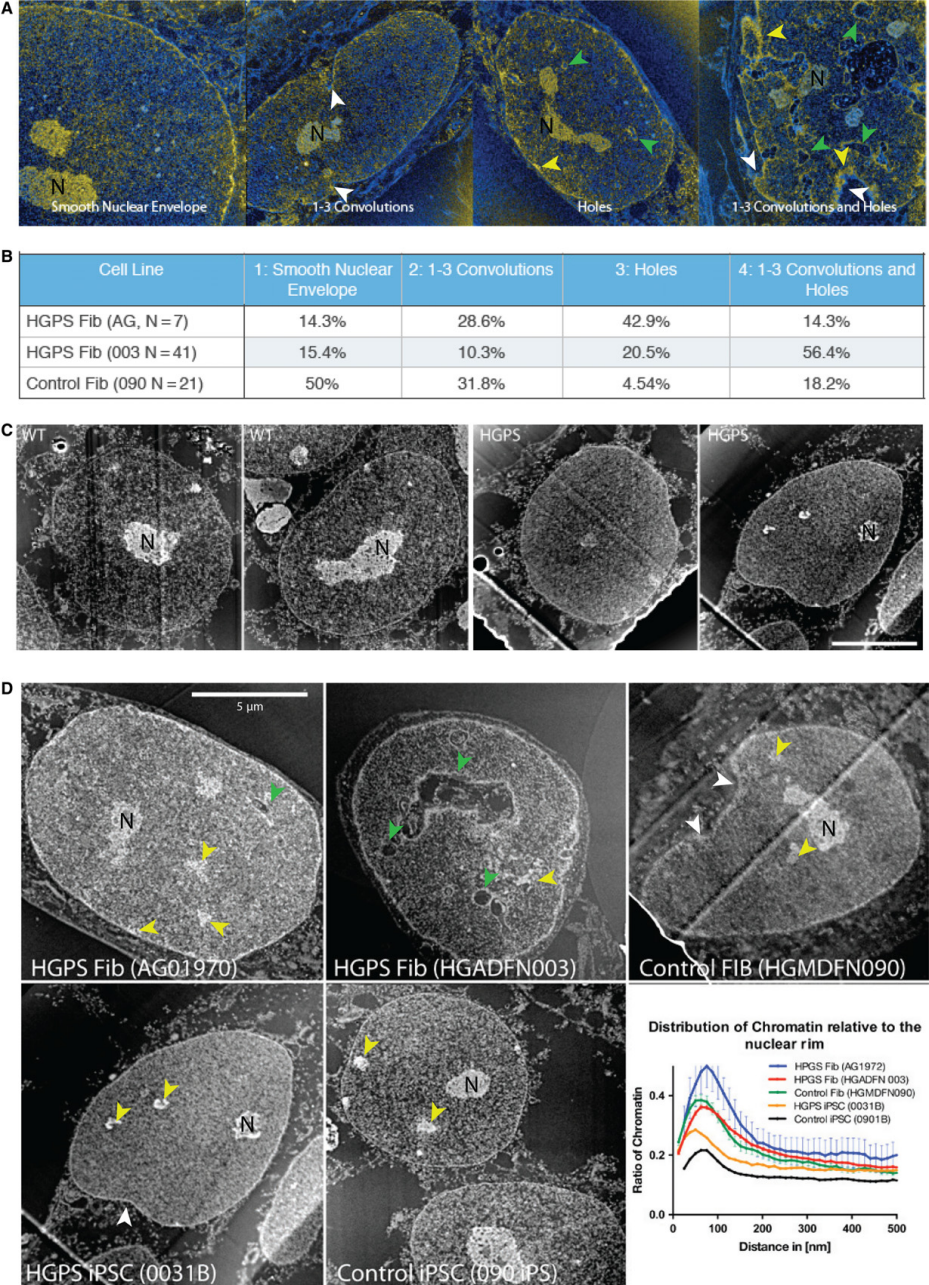
The distribution of nuclear phenotypes among patient and control fibroblasts and induced pluripotent stem cells (iPSCs) demonstrates significant rescue of nuclear defects by reprogramming. (A) Representative images illustrating the different nuclear morphologies that were scored. The images were obtained by combining phosphorus (yellow) and nitrogen (cyan) maps using electron spectroscopic imaging. Chromatin and nucleoli are the most prominent nucleic acid‐rich features (yellow) in the nuclei whereas ribosomes are the only structures enriched in nucleic acids in the cytoplasm, which otherwise only shows nitrogen‐rich protein structures (cyan). White arrows indicate convolutions and green arrows holes. N represents nucleolus. (B) Nuclei were evaluated for convolutions in the perimeter of the nucleus as well as regions of cytoplasm found within the cross‐section of the nucleus (holes), which reflects protrusions of cytoplasm in the *z*‐dimension. (C) Representative darkfield TEM images taken of control and Hutchinson–Gilford progeria syndrome (HGPS) iPSCs illustrating the morphology of the reprogrammed cell lines. (D) Chromatin of HGPS cells is remodeled to a normal pluripotent state by reprogramming. The images show 175 eV energy loss images (phosphorus enriched) where chromatin and nucleoli show enhanced contrast (white on a black background). The distribution of chromatin was quantified and plotted relative to the nuclear periphery. The *x*‐axis shows the periphery (leftmost part of graph) moving toward the center (rightmost part of the graph). The proportion of chromatin relative to total is plotted. The initial peak in the plot reflects the peripheral heterochromatin with higher values reflecting greater densities of heterochromatin associated with the nuclear lamina. White arrows indicate convolutions, green arrows holes, and yellow arrows condensed chromatin.

A striking feature of aged HGPS patient fibroblasts is the disruption of the morphology of the nuclear envelope, which becomes highly irregular (Fig. 3A). To examine defects in nuclear morphology, we performed electron spectroscopic imaging (ESI), an analytical and quantitative form of transmission electron microscopy (TEM; Bazett‐Jones & Hendzel, 1999; Bazett‐Jones *et al*., 1999). We grouped nuclei into four categories: (i) smooth nuclear envelope; (ii) three or less obvious convolutions or intrusions that disrupt the smooth perimeter; (iii) one or more intrusions of the cytoplasm into the cross‐section of the nucleus (i.e., holes), and (iv) both convolutions of the cytoplasm into the cross‐section and holes in the nucleus. We found that only about 15% of HGPS fibroblasts had smooth nuclear envelopes compared to half of control fibroblasts (Fig. 3B). However, these defects were absent in both normal and patient iPSC nuclei. Essentially all control (57/57 nuclei) and HGPS (30/31 nuclei) iPSC nuclei demonstrated normal nuclear morphology with smooth or smooth with minor undulations of the nuclear perimeter and showed no evidence of the HGPS nuclear phenotype (Fig. 3C). Thus, the defects that characterized the nuclear periphery in HGPS fibroblasts are restored to generate normal‐looking nuclei without obvious compromise of the nuclear periphery.

### Global expression profiling reveals a normal transcriptional landscape in undifferentiated HGPS iPSCs

The presence of Progerin in the nuclear lamina of HGPS fibroblasts is correlated with changes in global gene expression (Capell & Collins, 2006; Dechat *et al*., 2008). To determine whether reprogramming re‐establishes the normal transcriptional program that is characteristically misregulated in HGPS fibroblasts, we performed global mRNA expression analyses on all patient and normal fibroblast lines and their derived iPSC clones.

**Figure 4:**
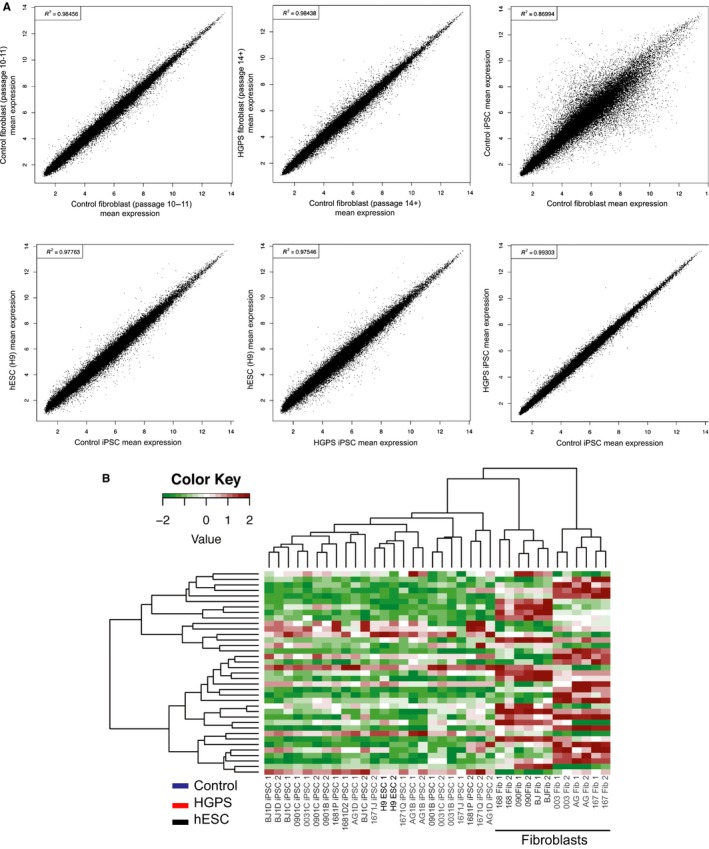
Global mRNA expression in Hutchinson–Gilford progeria syndrome (HGPS) fibroblasts correlates with control human embryonic stem cells (hESCs) and induced pluripotent stem cells (iPSCs). (A) mRNA microarray profiles of normal iPSCs and HGPS iPSCs were compared against profiles of hESCs (H9) and parental fibroblasts from controls or HGPS patients. Scatterplot analyses reveal that transcriptome profiles of HGPS iPSCs are highly similar to control iPSCs and hESCs, but significantly different from parental fibroblasts. (B) Hierarchical cluster analyses of all transcriptome profiles demonstrate that HGPS iPSCs cluster closely with hESCs and normal iPSCs, but are markedly different than parental fibroblasts from either normal or HGPS patients. The names of the cell lines are color‐coded to indicate the genotype: hESC lines, black; HGPS lines, red; and unaffected control lines, blue.

We first compared gene expression profiles between our normal fibroblasts and HGPS fibroblasts to characterize the extent of global gene expression changes in diseased cells. Specifically, we examined gene expression changes between the two groups at early (10–11 passages), corresponding to the initiation of the reprogramming process, and later (more than 14 passages) stages of their replicative span (Fig. 4A). Using unsupervised hierarchical clustering (not shown), we found the degree of dissimilarity between the expression profiles of the two groups increases from early to later passage, suggesting that global gene expression of Progeria fibroblasts becomes more dysregulated with each passage before they reach their replicative lifespan. We then assessed whether we could detect vestiges of the dysregulated transcriptomes in HGPS iPSCs. The global gene expression profiles between HGPS and control iPSCs indicate that the iPSC lines are much more similar to each other (Pearson correlation *r* ^2^ = 0.99) and to H9 hESCs (*r* = 0.98), and clustered more distantly from their parental fibroblasts (*r* = 0.88) (Fig. 4A).

Although no differentially expressed genes were identified by statistical analyses, we reasoned that patterns of expression across multiple genes might distinguish between control and HGPS iPSCs. To that end, we identified genes with the smallest *P*‐values as representing the best individual gene candidates. We then performed unsupervised hierarchical clustering of the reprogrammed control and HGPS samples, along with the unreprogrammed parental fibroblast samples (Fig. 4B). Hutchinson–Gilford progeria syndrome and control fibroblasts clearly clustered by genotype as shown in the right side of Fig. 4B. Not surprisingly, the biological replicates of each fibroblast cell line clustered together (adjacent leaves in the dendrogram). Furthermore, each branch represented distinct fibroblast cell lines. Similarly, biological replicates of the iPSC lines and different iPSC lines reprogrammed from the same parental fibroblasts also tend to cluster together (adjacent leaves), but not in every case. In fact, the iPSC lines did not always cluster by genotype, controls vs. HGPS iPSC samples did not definitively segregate, and some dendrogram branches included cell lines from both control and HGPS iPSCs. Similarly, the iPSCs originating from the trio of the parents (168 090) and the affected child (167) did not segregate in the clustering analysis nor by principle component analysis (PCA) (Fig. S2), showing that there were no differences between the related and unrelated controls and patients with HGPS in undifferentiated cells at the gene expression level. These results suggest that despite the presence of defects associated with Progerin in the nuclear lamina including structural defects, senescence, and abnormal epigenetic marks, HGPS fibroblasts can be reprogrammed into iPSCs with transcriptomes that are highly similar to control iPSCs and hESCs.

### Defects in heterochromatin distribution are rescued in HGPS iPSCs following reprogramming

**Figure 5:**
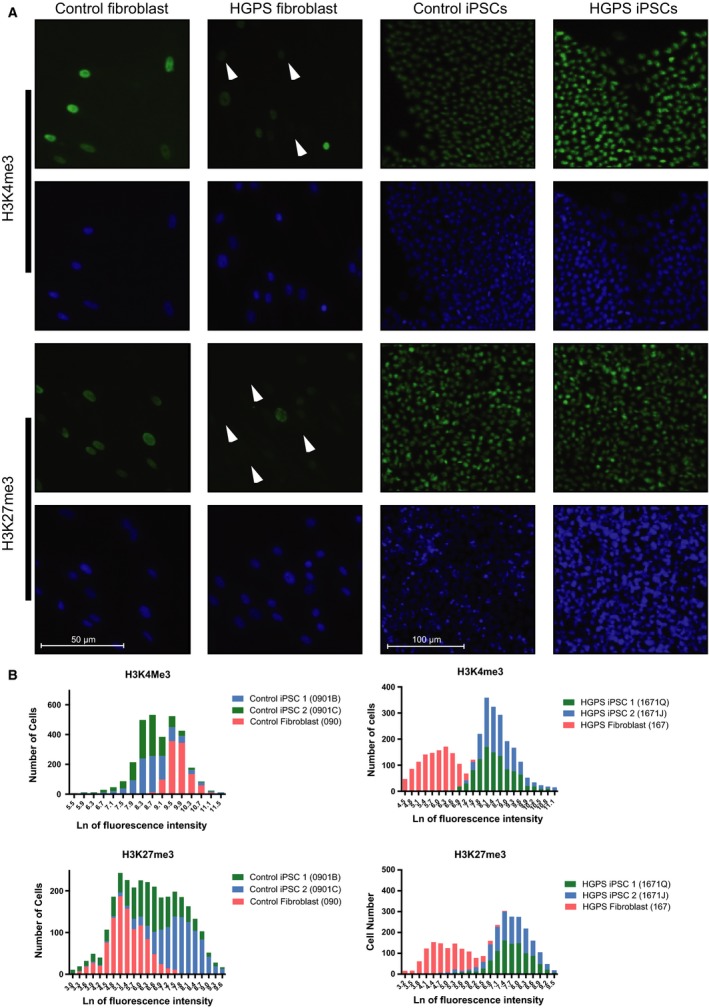
The epigenetic landscape is rescued following reprogramming. (A) High‐content imaging of histone marks H3K4me3 and H3K27me3 show a loss in Hutchinson–Gilford progeria syndrome (HGPS) fibroblast nuclei compared to normal fibroblasts. Arrows indicate nuclei with diminished H3K4me3 and H3K27me3 fluorescence signal. After reprogramming of HGPS induced pluripotent stem cells (iPSCs), H3K4me3 and H3K27me3 returned to levels comparable to normal iPSCs. (B) Intensity of H3K4me3 and H3K27me3 expression in fibroblasts (red bars) and corresponding iPSCs (green and blue bars) from normal and HGPS patients was quantified using high‐content imaging values compared on a natural log scale. Average intensities of both marks were lower in HGPS fibroblasts than normal fibroblasts, but following reprogramming, intensity levels returned to normal.

The loss of peripheral heterochromatin (Shumaker *et al*., 2006) and changes in epigenetic landscape, including the loss of the active histone mark H3K4me3 (Liu *et al*., 2013) and the repressive mark H3K27me3 (Shumaker *et al*., 2006; Shah *et al*., 2013), are defining characteristics of HGPS dermal fibroblasts. We sought to determine whether reprogramming rescued these epigenetic alterations by assessing the activation histone mark H3K4me3 and the repression histone mark H3K27me3. To measure the distribution of these two marks at the single cell level, we incorporated quantitative high‐content immunofluorescence imaging of our fibroblast and iPSC lines. The level of both marks was significantly diminished in the parental HGPS fibroblasts compared to the control fibroblasts (Fig. 5A,B). Some HGPS nuclei exhibited complete loss of both marks (Fig. 5A), which indicates significant chromatin perturbations. In contrast, the abundance of both marks in HGPS iPSCs is similar to those in control iPSCs. The levels of H3K4me3 and H3K27me3 were also much higher in normal or HGPS iPSCs compared to their respective parental fibroblasts.

Based on previous studies reporting the loss of peripheral heterochromatin in HGPS fibroblasts (Columbaro *et al*., 2005; Shumaker *et al*., 2006), we further inspected the distribution of heterochromatin in HGPS fibroblasts and iPSCs by ESI. We confirmed that irregular peripheral chromatin distributions (Fig. 3D) were common in HGPS fibroblasts relative to the control cells. We selectively imaged the chromatin by collecting maps of the distribution of phosphorus atoms within the specimen using ESI. From this, we evaluated the radial distribution of chromatin, which reflects its relative distribution from the nuclear lamina through to the center of the nucleus. Commonly, heterochromatin is enriched on the periphery of nuclei and this results in the largest concentrations of chromatin at the periphery. The radial distribution of chromatin was quantified and plotted. As expected, the HGPS and normal fibroblasts showed a strong bias toward chromatin accumulation near the periphery of the nucleus. Notably, the HGPS fibroblasts showed the greatest abundance at the surface, but this increase in chromatin density near the periphery was caused by the increased nuclear surface arising from significant nuclear invaginations, which were common in the HGPS fibroblasts but not as common nor as extreme in the normal fibroblasts.

Upon reprogramming, the nuclei take on a very different chromatin organization, with substantially fewer regions of obviously condensed chromatin (Fig. 3C). The chromatin is also observed to redistribute. Rather than accumulating chromatin near the periphery, the distribution of the reprogrammed cells is much more uniform, with very modest increased density near the periphery (Fig. 3D). This is evident by the near absence of a peak near the lamina (0–200 nm) in radial distribution plot. Taken together, this suggests that nuclear membrane disintegrity and irregular chromatin density in HGPS fibroblasts have reverted back to normal after reprogramming into iPSCs, similar to what we observed for gross nuclear morphology. The decondensed state of the chromatin is consistent with a less differentiated cell state.

### Undifferentiated HGPS iPSCs exhibit normal promoter proximal H3K4me3 and H3K27me3 profiles

**Figure 6:**
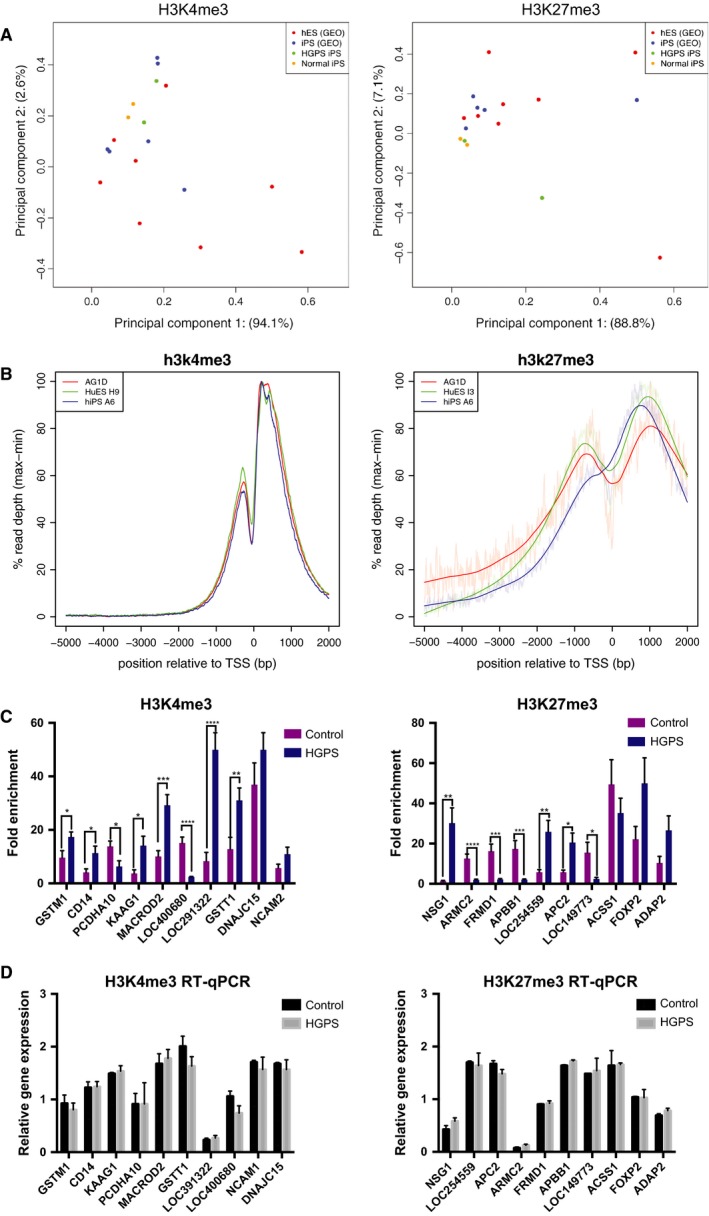
Undifferentiated Hutchinson–Gilford progeria syndrome (HGPS) induced pluripotent stem cells (iPSCs) exhibit normal promoter proximal H3K4me3 and H3K27me3 profiles. (A) Principle component analyses were performed based on H3K4me3 and H3K27me3 ChIP‐seq profiles of two HGPS (green) and two normal iPSC lines (yellow), which were then compared to publicly available hESC (red) and iPSC line data (blue). The iPSC lines described in this manuscript grouped largely with control hESC and iPSC lines with little variance. (B) The composite of average promoter proximal [−5 to +2 kb of the transcription start site (TSS)] reads of H3K4me3 and H3K27me3 for all RefSeq genes were compiled between the control and HGPS iPSC lines and compared to that of publicly available profiles of control hESC and iPSC cell lines. The H3K4me3 and H3K27me3 enrichment profiles were highly similar between all pluripotent cell lines, suggesting a normal epigenetic landscape in the HGPS iPSC lines. (C) A 50 kb sliding window to scan the ChIP‐seq data to discern genomic regions with differential enrichment between Progeria and control iPSCs identified 106 nonpromoter proximal differentially methylated regions associated with RefSeq genes across the whole genome. The top 10 most differentially enriched ChIP‐seq target genes were tested by quantitative ChIP‐PCR in all iPSC lines. Eight of 10 H3K4me3 and seven of 10 H3K27me3 marks were differentially enriched between normal and HGPS iPSCs, which are indicated by* (D) qRT–PCR of differentially enriched target genes was performed in all iPSC lines, which revealed no differences in gene expression between normal and HGPS iPSCs in any of the target genes. *indicates *P* ≤ 0.05, **indicates *P* ≤ 0.01, and ***indicates *P* ≤ 0.001.

Incomplete erasure of the cell of origin epigenetic signature has been previously noted in iPSC lines, leading to cell of origin transcriptional memory in iPSCs (Ohi *et al*., 2011) that has the potential to alter iPSC differentiation. An altered epigenetic landscape in HGPS iPSCs could affect analyses of differentiated iPSCs. Thus, to test whether the altered epigenetic state of HGPS fibroblasts was retained in iPSCs, we next investigated the extent of rescue of the epigenetic landscape by reprogramming. We performed ChIP‐seq on two HGPS iPSC clones and two unaffected iPSC clones to examine genomewide H3K4me3 and H3K27me3 profiles as a functional consequence of reprogramming (Fig. S4). To extend our dataset, PCA was performed between our iPSC data and publicly available hESC (BG01, WIBR1, WIBR3, HUES48, and HUES64) and normal iPSC (A1, C1, A6, 4, 18c, and 15b) H3K4me3 and H3K27me3 datasets. First, we focused our analyses within the −5 to +2 kb of the transcription start site region in all known coding genes. The reprogrammed HGPS iPSC lines grouped well with most of the publicly available hESC and iPSC data with little variance between the samples (Fig. 6A). Importantly, the heterogeneity in H3K4me3 and H3K27me3 ChIP‐seq profiles between our normal and HGPS iPSCs is less than the inherent heterogeneity within other groups of normal pluripotent cells (Fig. S3A,B). We also assembled a composite of average reads for all RefSeq genes between the iPSCs and compared it to the hESC H9 and iPSC A6 data and found that the enrichment profiles for H3K4me3 and H3K27me3 were highly similar between our iPSCs and that of the H9 and A6 profiles (Fig. 6B). Hence, reprogramming efficiently resets the H3K4me3 and H3K27me3 marks associated with the promotor proximal region, consistent with our finding that there are no statistically significant changes in gene expression between HGPS and control iPSCs.

To further extend our ChIP‐seq analysis beyond the promoter regions, we used a 50 kb sliding window to scan the ChIP‐seq data to identify genomic regions with differential enrichment between Progeria and control iPSCs using diffReps. This strategy identified 106 nonpromoter proximal regions (such as gene body and enhancer regions) associated with RefSeq genes that are differentially enriched for H3K4me3 and 38 regions for H3K27me3 (*P* < 0.01). We tested the top 10 differentially methylated regions for each histone mark in normal and HGPS iPSCs by ChIP‐qPCR; eight of 10 H3K4me3 and seven of 10 H3K27me3 marks were in fact differentially enriched between normal and HGPS iPSCs (Fig. 6C). Next, we compared any differentially H3K4me3‐ or H3K27me3‐enriched genes with our transcriptomic data and determined that none of the differentially methylated regions were associated with differential gene expression. To verify our bioinformatic analyses, we performed qRT–PCR for the same 20 genes (Fig. 6D) on normal and HGPS iPSCs. As expected from our microarray data, expression levels of these genes are not significantly different between normal and HGPS iPSCs, suggesting that the altered epigenetic marks outside of the promoter did not impact gene expression. Thus, our ChIP‐seq and transcriptional microarray analyses together support the hypothesis that the epigenetic landscape of HGPS fibroblasts has been reverted to a normal pluripotent state by reprogramming.

### HGPS and control VSMCs demonstrate comparable differentiation capability into VSMCs

**Figure 7:**
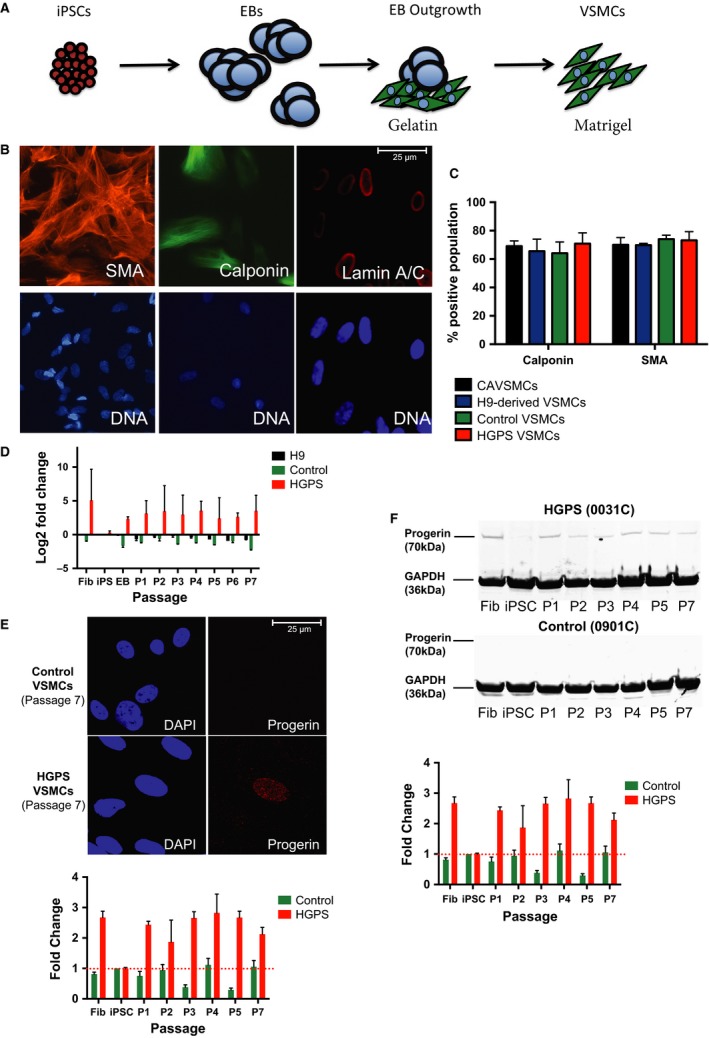
Direct differentiation of Progeria induced pluripotent stem cells (iPSCs) to vascular smooth muscle‐like cells. A directed differentiation method was employed to enrich for VSMCs. A schematic of VSMC differentiation protocol from iPSCs is outlined in (A) and utilizes a combination of different extracellular matrix proteins as well as specific vascular smooth muscle media to enrich for VSMC cells. (B) VSMCs expressed VSMC markers such as smooth muscle actin (SMA) and Calponin, while *LMNA* was upregulated. (C) Hutchinson–Gilford progeria syndrome (HGPS) iPSCs differentiated into VSMCs at the same efficiency as control iPSCs and H9 hESCs, exemplified by same percentage of cells expressing SMA and Calponin. Human coronary artery VSMCs (CAVSMCs) are shown as a positive control for SMA and Calponin expression. (D) *Progerin* transcript levels assessed by qPCR showed *Progerin* mRNA is detected in EBs and in each passage (P) of VSMCs, while no *Progerin* mRNA was detected in control iPSCs or H9 hESCs. (E) Confocal analysis Progerin‐positive cells in EB cultures, with increasing numbers in HGPS, but not control, VSMC cultures. (F) Western blot analysis of parental fibroblasts, undifferentiated (iPSC), EB outgrowth and various passages (P) of VSMC cultures. Progerin protein was not detected in HGPS iPSC cultures nor control cells but was detected in HGPS fibroblasts and VSMCs.

The real advantage of HGPS iPSCs is to differentiate patient cells into affected lineages to model HGPS. As patients with HGPS succumb to cardiovascular or cerebrovascular disease from the degeneration of vascular smooth muscle cells (VSMCs), we applied a directed differentiation strategy (Xie *et al*., 2007) to generate cultures of VSMCs (Fig. 7A). To assess the capacity of HGPS iPSCs to differentiate into VSMCs, we quantified differentiated cultures for VSMC‐specific markers Calponin and SMA (Fig. 7B). Control and HGPS iPSCs generated cultures expressing similar levels of Calponin (64% vs. 70%, respectively) and SMA (74% vs. 73%) (Fig. 7C), comparable to the expression of these markers by human coronary artery VSMCs and H9 hESC‐derived VSMCs (Fig. 7C). Finally, we verified that each VSMC culture responds physiologically in response to the cholinergic agonist carbachol (data not shown). Thus, HGPS iPSCs readily differentiate into VSMCs, an affected cell lineage.

Next, we analyzed the expression of Progerin in iPSCs differentiated into VSMCs early in the differentiation process (EB) and across several passages of VSMCs to determine whether we could model disease initiation and its progression *in vitro*. qRT–PCR analysis revealed that in HGPS cultures, *Progerin* mRNA increases dramatically compared to undifferentiated iPSCs, detected as early as the EB stage of differentiation and in VSMC cultures including passage (P) 1 (Fig. 7D). As expected, *Progerin* mRNA expression is negligible in H9 hESC‐ and control iPSC‐derived cultures. Confocal imaging of differentiating EB cultures identified approximately 2% of HGPS EB cells contained detectable levels of Progerin, while 10% of HGPS cells in passage 1 VSMC cultures demonstrated detectable levels of Progerin (Fig. 7E). By passage 4, more than 20% of HGPS VSMCs demonstrated detectable levels of Progerin, while differentiated iPSC control cultures showed no Progerin expression. Similarly, Western blot analysis failed to detect Progerin in undifferentiated HGPS iPSCs, while Progerin protein levels were detectable by passage 1 of HGPS VSMC cultures (Fig. 7F). These results suggest that Progerin expression starts shortly after differentiation from pluripotent stem cells and increases in VSMCs, suggesting that iPSC‐derived VSMCs can be used to model the initiation and progression of the molecular consequences of Progerin accumulation.

### HGPS VSMCs exhibit impaired DNA damage repair response

**Figure 8:**
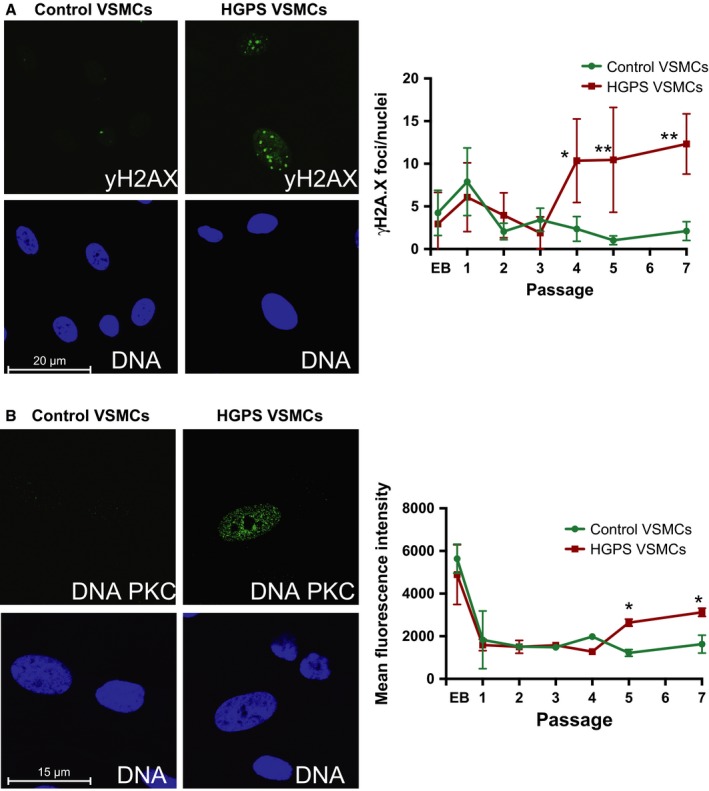
Hutchinson–Gilford progeria syndrome (HGPS) VSMCs exhibit increased DNA double‐strand breaks. Confocal analysis of DNA damage response markers in VSMC differentiation cultures (EBs and various passages (P) of VSMCs. (A) γH2A.X foci are equivalent between control and HGPS cultures until passage 4 of VSMC cultures. (B) Increase in DNA‐PKC accumulation in HGPS VSMCs was detected by Passage 5. *indicate *P* ≤ 0.05, **indicate *P* ≤ 0.01.

Hutchinson–Gilford progeria syndrome cells accumulate endogenous DNA damage, in particular double‐strand breaks (DSBs), and exhibit impaired DDR with passage in culture (Liu *et al*., 2005, 2006, 2008). As persistent DSB repair could result in epigenetic remodeling, we focused on characterizing DSBs in HGPS VSMCs, which are indicated by the presence of cryptogenic foci marked by the phosphorylated histone H2A.X (γH2A.X). Using γH2A.X immunostaining to detect cryptogenic foci at each passage throughout the differentiation process, we observed an elevation in γH2A.X foci in both control and HGPS VSMCs in their EB outgrowth stage and at passage 1 (Fig. 8A). This level of γH2A.X foci in these cultures may be reflective of the activation of DDR signaling cascade that occurs during cell differentiation processes (Sherman *et al*., 2011). While both control and HGPS VSMCs exhibited reduced γH2A.X foci at passages 2 and 3, HGPS VSMCs exhibited a dramatic increase in γH2A.X foci level beginning at passage 4, whereas control VSMCs demonstrated a persistently low γH2A.X level following passage 2 (Fig. 8A). To identify the source of increased γH2A.X, we monitored the expression of DNA‐dependent protein kinase catalytic subunit (DNA‐PKC) in HGPS VSMCs, as the phosphorylation of H2A.X by DNA‐PKC is required for the DDR activation (Urushihara *et al*., 2012). Using high‐content imaging, we found a significant increase in DNA‐PKC fluorescence intensity in HGPS VSMC nuclei starting at passage 5 compared to normal VSMCs (Fig. 8B), suggesting that DDR is persistently activated due to high levels of DNA damage, and this activation occurs a few passages following Progerin accumulation (Fig. 7E). Overall, our differentiation data suggest that HGPS iPSCs can differentiate into VSMCs with no initial phenotypes, and can be used to monitor the initiation and progression of molecular and cellular phenotypes.

## Discussion

Hutchinson–Gilford progeria syndrome encompasses a spectrum of accelerated aging features and is considered the most severe form of Progeria. Hutchinson–Gilford progeria syndrome patient‐derived dermal fibroblasts demonstrate increased nuclear deformity and blebbing, DNA damage, senescence, and loss of heterochromatin (Csoka *et al*., 2004; Scaffidi & Misteli, 2008). Recently, several reports described the generation of iPSCs derived from HGPS fibroblasts (Liu *et al*., 2011a,b; Zhang *et al*., 2011, 2014; Nissan *et al*., 2012). Although some of these reports have confirmed the iPSC‐derived differentiated cells recapitulate some of the hallmarks of HGPS and provided some molecular insight to the mechanisms involved in HGPS, these studies lack structural and genomewide characterization of the epigenetic landscape in patient‐derived iPSC lines. Therefore, the question that remained is whether reprogramming of HGPS fibroblasts erases the Progerin‐induced senescent epigenetic landscape and nuclear defects, resulting in truly normal iPSCs that will allow the analysis of HGPS from initiation through disease progression. Despite the initial compromised state of patient fibroblasts, HGPS iPSCs are pluripotent, with baseline morphological, transcriptional, and epigenetic profiles comparable to that of normal iPSCs and hESCs. The phenomenal power of reprogramming is thus able to reset the epigenetic landscape and overcome significant phenotypic impairments to create pluripotent stem cells that are highly similar to normal stem cells.

We systematically tested for vestiges of HGPS phenotypes in our iPSCs to assess the reprogramming potential of these cells. Firstly, we considered nuclear defects. In HGPS cells, the nuclear lamina is highly disorganized. Typically, the accumulation of Progerin provokes a severe distortion of the nuclear envelope with significant loss of heterochromatin compartmentalization (Scaffidi & Misteli, 2005; Shumaker *et al*., 2006). We observed many deep membrane invaginations in the nuclear periphery within HGPS fibroblasts and irregular chromatin distribution around invaginations. It is evident that the nuclear lamina is required for the correct organization of heterochromatin in the nuclear periphery, and the accumulation of Progerin strongly interferes with nuclear architecture and chromatin spatial distribution. However, reprogrammed HGPS iPSCs exhibited none of the gross morphological defects associated with the HGPS fibroblasts used in the reprogramming experiments; the iPSCs displayed an overall smooth nuclear perimeter or had minor undulations identical to what can be seen in control iPSCs and hESCs, demonstrating the silencing of Lamin A and Progerin in our HGPS iPSCs via reprogramming has rescued the nuclear architecture defects.

The nuclear lamina and lamins provide a repressive environment for gene expression due to specific chromatin–lamin interactions (Lammerding *et al*., 2004; Favale *et al*., 2007; Peric‐Hupkes *et al*., 2010; Puckelwartz *et al*., 2011). Therefore, it is believed that transcriptional dysregulation in HGPS is due to the disruption of lamina network integrity by Progerin accumulation (Taimen *et al*., 2009). Our microarray analyses revealed that although HGPS fibroblasts differ from unaffected fibroblasts, gene expression profiles are common between our HGPS iPSCs and normal iPSCs, and using multiple forms of analysis and statistical rigor, no differentially expressed genes were identified between the two groups. This demonstrates that undifferentiated HGPS iPSCs transcriptionally resemble control pluripotent cells despite the defects in the donor fibroblasts including aberrant epigenetic and transcriptional landscape.

Profound alterations in histone covalent modifications and epigenetic landscape of HGPS fibroblasts have been widely reported. Loss of peripheral heterochromatin and various histone modifications are commonly observed in HGPS fibroblasts (Goldman *et al*., 2004; Shumaker *et al*., 2006). H3K27me3 is a repressive chromatin mark and is typically downregulated in HGPS cells. On the other hand, H3K4me3 is a histone mark associated with promoters of transcriptionally active genes, and was shown to be dysregulated in HGPS cells as well. We observed a loss of both histone marks in HGPS fibroblasts. These aberrant levels of H3K4me3 and H3K27me3 were restored to normal in all iPSCs following reprogramming. Genome‐wide enrichment profiles of both marks showed little variance between HGPS and normal iPSCs, further confirming that the epigenome in our iPSC clones was restored to normal by reprogramming. The few differentially methylated regions in HGPS iPSCs were found outside gene promoters and had no effect on overall gene expression in the undifferentiated cells.

HGPS cells in culture exhibit profound DNA damage and impaired activation of DDR signaling cascade, and overall increased genome instability in culture (Liu *et al*., 2005, 2006, 2008; Richards *et al*., 2011). Along with an increase in dysmorphic nuclei, HGPS cells also exhibit an increase in cryptogenic foci, contributing to the premature aging phenotype. As the most significant clinical feature of HGPS is the vascular deterioration, we differentiated HGPS iPSCs into VSMCs to analyze DSBs. Early in the differentiation cultures, γH2A.X and DNA‐PKC were elevated in both control and HGPS cells, consistent with the activation of DDR signaling that occurs during cell differentiation (Sherman *et al*., 2011). However, as the VSMC cultures were passaged, not only did we observe gradual Progerin accumulation following differentiation, we found higher levels of cryptogenic foci marked by γH2A.X, as well as an increased expression of DNA‐PKC in HGPS VSMCs compared to controls. Overall, our analyses demonstrate that the HGPS iPSCs reported here can be differentiated into relevant cell types to analyze disease initiation and progression.

Having demonstrated that these HGPS iPSCs revert to a normal pluripotent identity and are capable of differentiating into an affected lineage reflecting disease phenotype, we propose that the patient/control‐matched library of cells will serve a valuable resource for the field of aging research. iPSCs provide a virtually limitless source of cells, which can then be differentiated into cells of choice to study tissue‐specific aspects of disease progression. Additionally, a cell source with unlimited differentiation potential allows tissue‐specific drug screens to identify novel therapeutics. The iPSC lines derived from healthy individuals, including parents of one proband, provide excellent controls for future studies. Beyond investigating the pathology behind HGPS, the iPSC panel provides a resource that could be used to study tissue‐specific pathology during the normative aging process. Specifically, these HGPS iPSCs with normal nuclear structure provide an excellent platform to understand the direct and indirect consequences of altered associations between heterochromatin and the nuclear lamina.

In summary, our data demonstrate that despite the compromised state in donor HGPS fibroblasts, the morphological nuclear defects and the abnormal transcriptional and epigenetic landscape were rescued in the reprogramming process and resulting iPSCs. These were critical findings as our ultimate goal is to use a systems genetics strategy (Chang *et al*., 2012) to dissect the initiation and progression of HGPS in affected and unaffected cell lineages derived from iPSCs. This resource can also be used to uncover the mechanisms underlying altered epigenetic inheritance in Progeria and aging. While the undifferentiated iPSC lines lack differentially regulated genes between the HGPS and controls despite some differential H3K27me3 and H3K4me3 methylation outside of the promoters between samples, it will be important to determine whether aberrant gene expression is associated with these methylated regions upon differentiation into affected and unaffected cell lineages. Similarly, there were no differences between the related and unrelated controls and patients with HGPS in undifferentiated cells at the gene expression level; however, it will be interesting to determine whether alterations in gene expression and epigenetic marks will cluster differentially between the trio and other individuals in differentiated cell types.

## Experimental procedures

### Resources generated in this study

The iPSC lines 0901B, 0901C, 1681D2, 1681P, 0031B, 0031C, 1671J, 1671Q generated from fibroblasts from the Progeria Research Foundation biobank are available via the Progeria Research Foundation through a Materials Transfer Agreement, while BJ1C, BJ1D, AG1B, AG1D may be obtained from Coriell, the supplier of BJ and AG01972 fibroblasts. The transcriptomic and ChIP‐seq datasets are publicly available, deposited in the GEO database (accession numbers: to be activated upon acceptance of manuscript).

### Reprogramming

Retrovirus was generated using constructs pMXs‐hOCT4, pMXs‐hSOX2, pMXs‐hKLF4, pMXs‐hc‐MYC (Addgene) as described previously (Hotta *et al*., 2009; Chang *et al*., 2013). To generate VSV‐G pseudotyped retrovirus, Plat‐GP cells were transfected with 15 mg of expression vector and 5 mg of pVSV‐G. Two days post‐transfection, retrovirus was collected and filtered through a 0.45‐mm filter. Transduction of 5 × 10^5^ patient fibroblasts was performed by the addition of polybrene to a final concentration of 4 mg mL^−1^ to a retroviral cocktail either containing a combination of four factors or five factors. After transduction, fibroblasts were maintained in fibroblast medium for 6 days, then trypsinized and replated onto fresh mouse embryonic fibroblasts (MEFs). Fibroblast media was then replaced with hESC media and was changed daily. Approximately 20 days later, colonies resembling hESCs in morphology were mechanically picked and replated onto fresh MEFs. These iPSCs were mechanically dissociated for a few passages and then adapted to collagenase IV passaging.

### Cell culture

H9 hESCs and iPSCs were maintained on Matrigel in E8 medium (DMEM/F12 supplemented with l‐ascorbic acid, sodium selenite, bFGF, TGF‐β1, sodium bicarbonate, holo‐transferrin, and gentamycin). Hutchinson–Gilford progeria syndrome and normal patient fibroblasts were obtained from Progeria Foundation and Coriell, and were maintained in fibroblast medium (DMEM supplemented with 10% FBS, glutamax, and gentamycin). VSMCs were maintained in Medium 231 (Life Technologies, Canada) supplemented with VSMC growth supplement (Life Technologies).

### Vascular smooth muscle‐like cell differentiation

Differentiation of hESCs and iPSCs to VSMCs was essentially conducted as described by Xie *et al*. (2007) with minor modifications. Briefly, EBs were generated as described above and cultured in suspension in low cluster plates. After 7 days, EBs were plated on 0.1% gelatin‐coated plates for 3–5 days. EB outgrowths were trypsinized and cells were replated on Matrigel in Medium 231 (with growth supplement, Life Technologies). To differentiate VSMCs, cells were plated on gelatin‐coated six‐well plates and grown in VSMC differentiation medium (Medium 231, differentiation supplement, Life Technologies) for 7 days and characterized at each passage.

### Immunofluorescence, flow cytometry, high‐content imaging

Immunofluorescence analyses were performed as previously described (Chang *et al*., 2004). Briefly, cells were fixed in formalin for 15 min at room temperature and washed with PBS. Samples were then permeabilized with 0.1% Triton X‐100 for 20 min and blocked for 1 h with 3% skim milk in PBS, and primary antibodies were incubated for 2 h at room temperature or overnight at 4 °C. Primary antibodies used were Tra‐1‐60, Tra‐1‐81, SSEA‐4, Lamin A/C, HP1γ, Progerin (Millipore, USA), H3K4me3, H3K27me3 (Abcam, USA), γ‐H2A.X (New England Biolabs, USA), and DNA‐PKC (Abcam). High‐content imaging was performed essentially as previously described (Walker *et al*., 2007, 2010). Target activation algorithm and unbiased quantification analyses were incorporated for quantifying fluorescent signals in each image.

### Western blot

Whole‐cell lysates were prepared by dissolving cells in cell lysis buffer (50 mm Tris–HCl, 150 mm NaCl, 1 mm EDTA, 10% glycerol, 1% Triton X‐100) and sonicated for 10 s. Cell lysates were mixed with Laemmli sample buffer containing 2‐mercaptoethanol (Life Technologies) and heated at 70 °C for 10 min before loading. Blots were blocked with 5% milk and probed for Progerin (Millipore) and GAPDH (Abcam) as loading control. Band density was analyzed using ImageJ (NIH, USA).

### qPCR

Quantitative PCR was performed as previously described (Walker *et al*., 2007). Serial dilution qPCR primers that were used in this report have been previously reported to detect Progerin mRNA transcripts (Scaffidi & Misteli, 2006).

### Embryoid body (EB) and teratoma assays

To assess germ layer differentiation *in vitro*, EBs were generated from hESCs and iPSCs by treating with collagenase IV for 30 min at 37 °C and gently dislodged with a cell scraper, followed by plating in low cluster plate (Corning, USA) for 7–10 days in EB medium (DMEM/F12 supplemented with 20% knockout serum, glutamax, β‐mercaptoethanol, nonessential amino acids, and gentamycin). EB outgrowths were stained with antibodies against βIII‐tubulin (Millipore), SMA (Millipore), and Gata 4 (Santa Cruz, USA) to represent all three germ layers. For teratoma assays, three wells of a six‐well plate iPSC culture at 70–80% confluency were treated with collagenase for 30 min. The cells were isolated with a cell scraper, pelleted, and resuspended in 50% Matrigel and injected intramuscularly into NOD/SCID mice. After 8–10 weeks, tumors were removed, fixed in formalin, and sectioned with hematoxylin and eosin staining.

### Microarray analyses

Total RNA was isolated from cell pellets using the Nucleospin^®^ RNA kit (Machery‐Nagel, USA) according to the manufacturer's instructions. Integrity and quantity of the isolated RNA were measured using the Bioanalyzer RNA 6000 nanochip (Agilent, USA). Three hundred nanogram of total RNA was labeled and hybridized to GeneChip Human Gene 2.0 ST arrays (Affymetrix, Canada) according to the manufacturer's protocol and scanned on a GeneChip Scanner 3000 7G (Affymetrix, Canada). Two replicates were performed for each sample. Gene expression data from Affymetrix HuGene 2.0 microarrays were normalized using the RMA function of the R oligo package. Quality analysis of the microarrays was performed using the R arrayQualityMetrics package. Fold change and significance for transcript cluster identifiers between conditions were determined using the R limma package and annotated using the hugene20sttranscriptcluster.db (v2.14.0).

### ChIP‐seq

ChIP was performed as previously described (Chang *et al*., 2006) with some modifications. Briefly, nuclear pellets were resuspended in TC13 tubes (Covaris) with 2 mL of lysis buffer 3 (10 mm Tris–HCl pH 8, 100 mm NaCl, 1 mm EDTA, 0.5 mm EGTA, 0.1% Na‐deoxycholate, 0.5% Na‐lauroylsarcosine) and sheared using a Covaris S2 sonicator (20% duty cycle, intensity 5, 200 cycles/burst) for 9 min per sample. Three hundred microgram of chromatin was used for each sample. Before addition of antibodies, an aliquot of each sample was reserved for input control. Antibodies used were as follows: rabbit H3K4Me3 (Millipore), rabbit H3K27Me3 (Cell signaling Technology, USA), and normal rabbit IgG (Cell Signaling Technology). After reverse cross‐linking and cleanup, approximately 20 ng of each sample was prepared for sequencing using the ChIP‐seq sample rep kit (Illumina) according to the manufacturer's instructions. Samples were sequenced on an Illumina GAIIx (Illumina).

Alignment of ChIP‐seq to the human genome (hg19) data was performed using bowtie 1.1.0 with the following parameters: “‐l 32 –strata –best ‐y –chunkmbs 3072 ‐n 2 ‐m 1”. Duplicate reads were removed using the RMDUP function of samtools. In the case of SRA format files from the Short Reads Archive, the NCBI SRA toolkit versions 2.4.3 (NIH, USA) was used to convert the files for alignment with the above parameters. Known human genes were downloaded from ENSEMBL 74 to determine gene ENSEMBL identifiers, symbols, gene start, gene end strand, and gene biotype. Analysis was limited to genes on standard chromosomes (1‐23,X,Y).

DiffReps (Shen *et al*., 2013) was used to identify genomic regions differentially enriched for H3K27me3 and H3K4me3. ChIP‐seq BAM files reads were converted to BED files. Fragment lengths used for input into BIDCHIPS were the average of the fragment lengths of the treatment and control files as estimated by MaSC (Ramachandran *et al*., 2013). The different histones were analyzed with parameters as recommended in the paper. H3K4me3 used the —nsd ‘sharp’ setting and the ‘peak’ mode as it has sharper more localized enrichment, while H3K27me3 used the “—nsd” ‘broad’ setting and ‘block’ mode as it has broader more distributed enrichment. The ‘broad’ mode uses a 10‐kb window and step size while the ‘peak’ setting uses a 1‐kb window and step size. The resulting output identifies windows in the genome where the histone reads are significantly different between treatment and control, taking into account the IgG background.

For PCA analyses and pileups, known protein‐coding genes, reads in the region −5 kb/+2 kb around the gene start were counted from H3K4me3, and reads over the full gene were counted for H3K27me3. The *r prcomc* function was used to determine the first two principal components, which were then plotted. Depths of reads for each data set for the regions around gene starts were determined to generate the pileup plots.

### ChIP‐qPCR

ChIP was performed as previously described with slight modifications (Walker *et al*., 2010). 500 000 iPS cells were sheared using Covaris S2 sonicator for 10 min before incubated with Magnetic A beads (Millipore) with H3K4Me3 or H3K27Me3 antibody overnight. 10% of the pull‐down was saved as input. Pull‐downs were reverse‐crosslinked using Eppendorf Thermomixer for 15 min and precipitated using phenol–chloroform. DNA quantity was measured using the Bioanalyzer RNA 6000 nanochip (Agilent). qPCR was performed as described above and repeated three times for each gene.

### Sample preparation for transmission electron microscopy (TEM) and electron spectroscopic imaging (ESI)

Cells were cultured in 35‐mm MatTek glass‐bottom dishes and fixed with 4% paraformaldehyde, 1 × phosphate‐buffered saline (pH 7.5) for 30 min. Postfixation was carried out using 2% glutaraldehyde, 1 × PBS for 30 min, and 0.5% OsO_4_ for 20 min. After a dehydration series (30%, 50%, 70%, 90%, 100% ethanol), cells were infiltrated with LR‐White^®^ Resin (LR‐White^®^‐100% ethanol 1:1 4 h and 100% LR‐White^®^ resin 4 h) before polymerizing the resin as described in Strickfaden *et al*. (2015). Appropriate areas with cells were identified on the cured blocks using a stereo microscope, and 50‐nm‐thin sections of the cells were cut with an ultramicrotome (Leica EM UC6) collected on 300‐mesh copper grids. Grids were carbon coated with a thin layer of carbon (< 1 nm) in a Cressington 208Carbon. ESI was performed on a JEOL 2100F 200 kV TEM equipped with a LaB6 electron source and a Gatan Tridiem GIF energy filter. Phosphorous ratio maps were acquired using the following settings: postedge collected at 175 eV electron energy loss with slit width of 20 eV; pre‐edge collected at 110 eV electron energy loss with a slit width of 20 eV. Nitrogen ratio maps were acquired using the following settings: postedge collected at 447 eV electron energy loss with a slit width of 35 eV; pre‐edge collected at 358 eV energy loss with a slit width of 35 eV. Images were processed as previously described in Strickfaden *et al*. (2015).

### Morphological grading of the cell nuclei

Nuclei were graded and assigned to one of the following categories: (i) smooth nuclear envelope, (ii) 1–3 convolutions, (iii) holes, and (iv) convolutions and holes.

### Measuring the chromatin density at the nuclear periphery

Phosphorous ratio maps of whole nuclei were treated as follows: images were converted into 8‐bit and normalized in ImageJ. The background was fitted and removed in Adobe Photoshop using plugins from the FoveaPro4 toolkit for background fitting and background removal. To create a mask, the outline around each nucleus was manually drawn in ImageJ. The Euclidian distance maps (EDM) were generated from these masks. Automatic thresholding was applied to the images in order to create binary images. The binary image was multiplied with the EDM. The histogram of the resulting image represents the absolute amount of chromatin relative to a distance from the nuclear rim. To normalize these data, the histograms were divided by the histogram of the EDM. These relative densities were averaged over all available nuclei of each cell line and plotted in Prism 6.0.

### Statistical analysis

Prism 6.0 was used for statistical analysis as described. All values were presented as means ± SEM unless stated otherwise. Two‐tailed unpaired *t‐*tests were performed to evaluate the difference between two groups of data. *indicates *P* value < 0.05 measured with Student's *t*‐test.

## Conflict of interest

None declared.

## Funding

Canadian Institutes of Health Research (Grant/Award Number: MOP‐133570), Progeria Research Foundation (Grant/Award Number: PRF‐009‐003), Heart & Stoke Foundation of Canada (Grant/Award Number: G‐13‐0003053).

## Author contribution

ZC, WYC, MJH, and WLS designed the study and interpreted the results. ZC and WYC conducted experiments, analyzed data and wrote the manuscript along with WLS. AE and HS performed experiments and interpreted results, while ZJ, SYK, CD, and AR assisted in conducting experiments and analyzing data. GAP, J‐HC, KW, and TJP performed bioinformatic analyses. AH, JE, RAK, FJD, and DJG provided reagents and helped interpret data. All authors assisted in editing the manuscript.

## Supporting information


**Fig. S1** iPSCs derived from normal patient fibroblasts are pluripotent.

**Fig. S2** Principle component analysis (PCA) of iPSC microarray profiles of familial trio (father HGFDN168, mother HGMDFN090 and affected son HGDFN167) compared to control iPSCs (BJ1) (A) and unrelated HGPS iPSCs (AG01972) (B).

**Fig. S3** Progeria iPSCs exhibited normal expression of chromatin‐associated histone marks H3K4me3 and H3K27me3.

**Fig. S4** Genome tracks of ChIP‐seq data for H3K4me3 and H3K27me3 of two representative differentially‐enriched genes in normal and HGPS iPSCs.
