# Supplementary figures and images for: Reprogramming progeria fibroblasts re‐establishes a normal epigenetic landscape
Source: Aging Cell. 2017 Jun 8;16(4):870–87. doi: 10.1111/acel.12621 (PMC5506428; doi:10.1111/acel.12621)

Supplemental Figure 1.

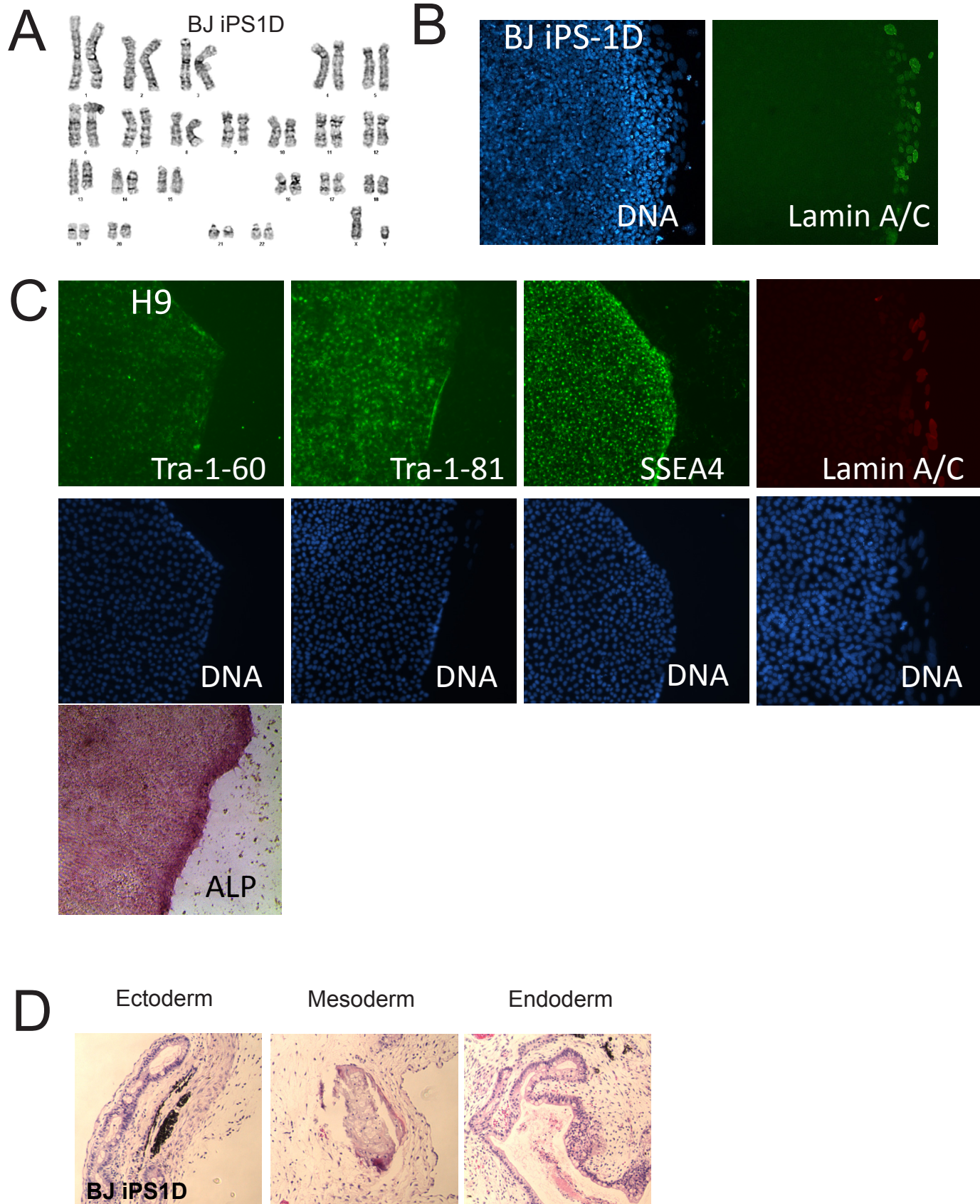

Supplement: Supplementary file 1 — Fig. S1 iPSCs derived from normal patient fibroblasts are pluripotent. [file ACEL-16-870-s001.pdf]

Supplemental Figure 2.

A

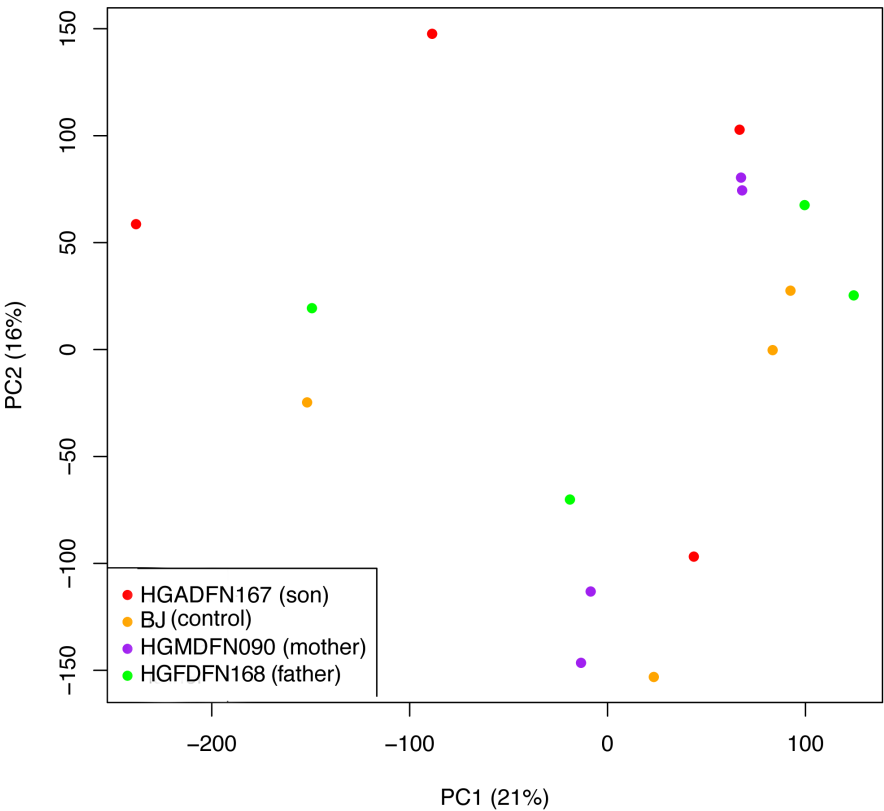

B

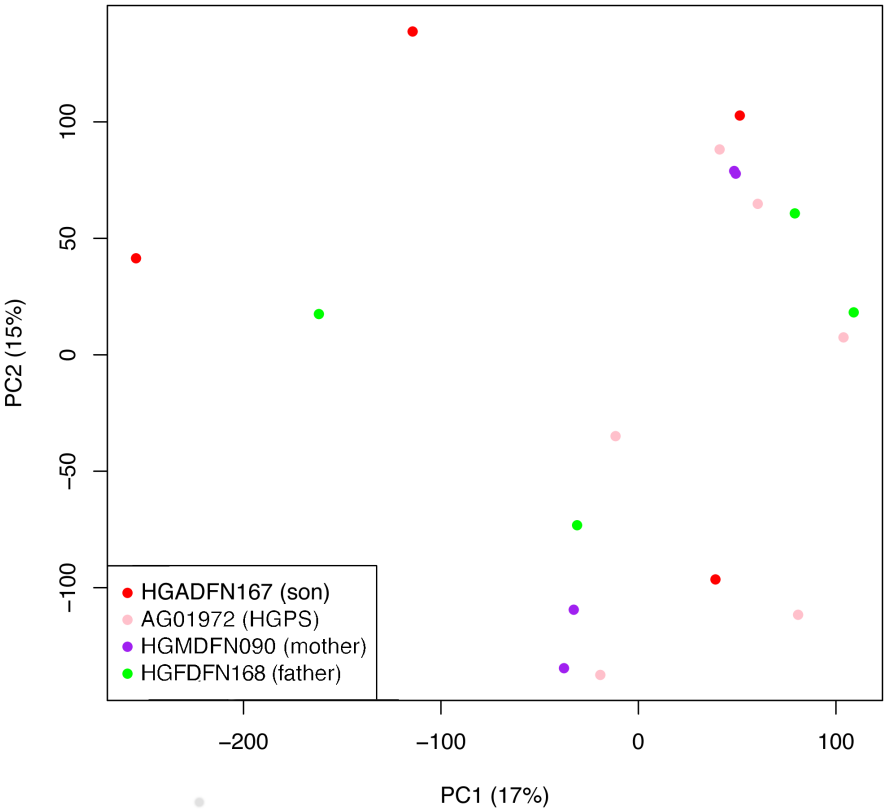

Supplement: Supplementary file 2 — Fig. S2 Principle component analysis (PCA) of iPSC microarray profiles of familial trio (father HGFDN168, mother HGMDFN090 and affected son HGDFN167) compared to control iPSCs (BJ1) (A) and unrelated HGPS iPSCs (AG01972) (B). [file ACEL-16-870-s002.pdf]

Supplemental Figure 3.

A

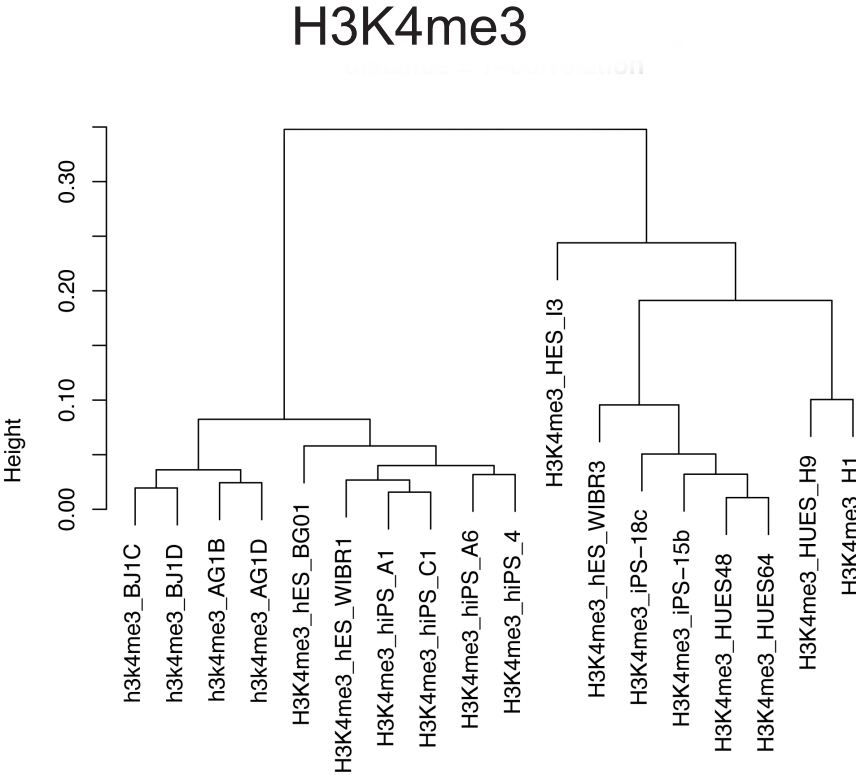

B

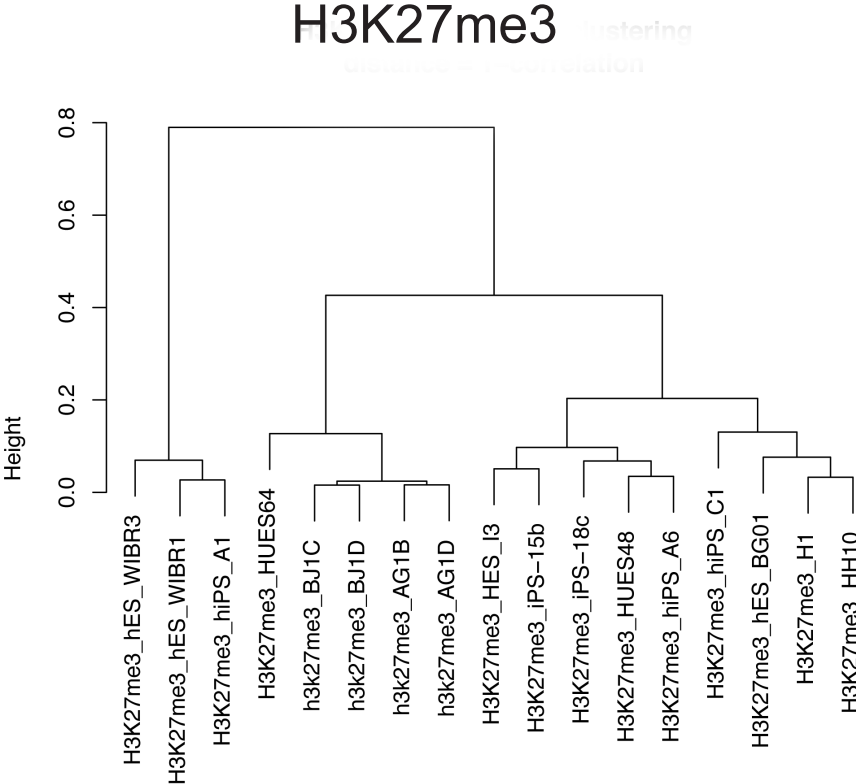

Supplement: Supplementary file 3 — Fig. S3 Progeria iPSCs exhibited normal expression of chromatin‐associated histone marks H3K4me3 and H3K27me3. [file ACEL-16-870-s003.pdf]

Supplemental Figure 4.

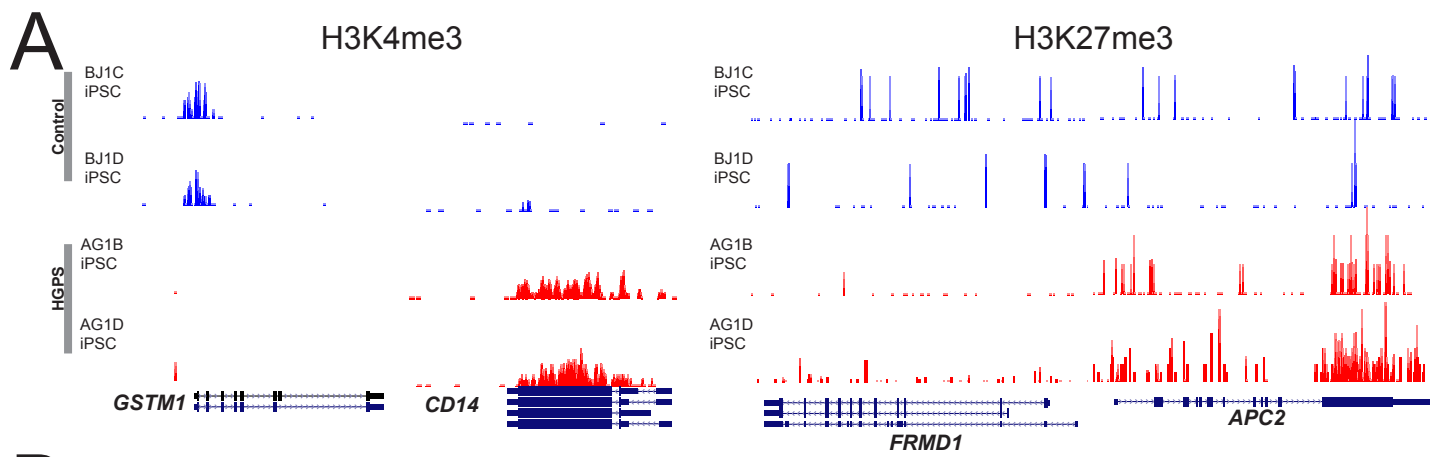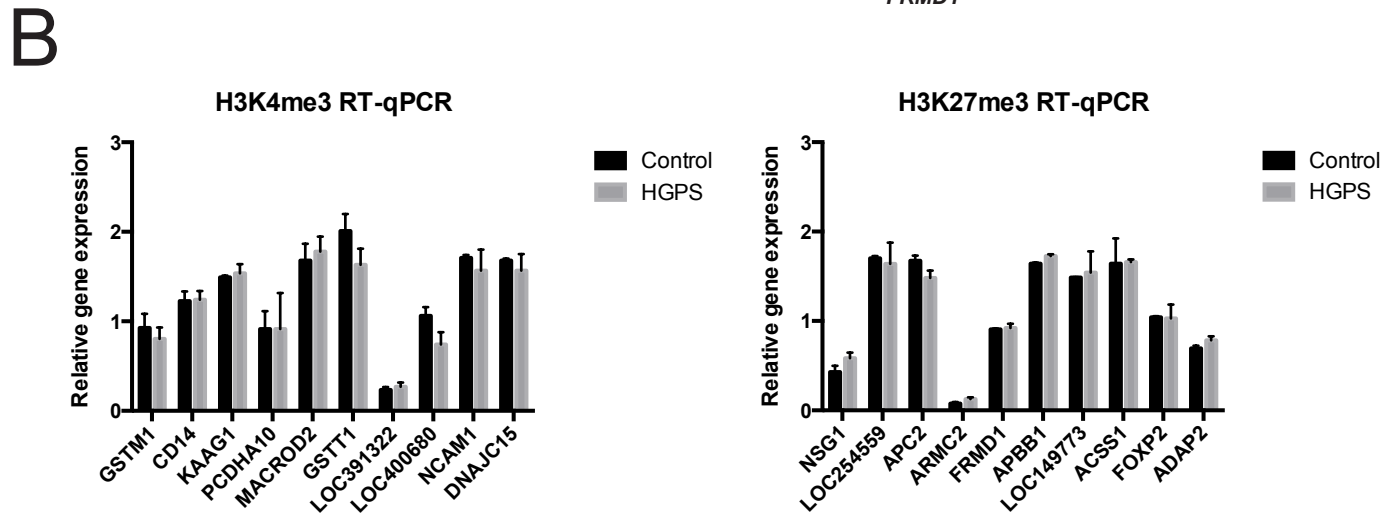

Supplement: Supplementary file 4 — Fig. S4 Genome tracks of ChIP‐seq data for H3K4me3 and H3K27me3 of two representative differentially‐enriched genes in normal and HGPS iPSCs. [file ACEL-16-870-s004.pdf]
